# From dawn to dusk—mimicking natural daylight exposure improves circadian rhythm entrainment in patients with severe brain injury

**DOI:** 10.1093/sleep/zsac065

**Published:** 2022-03-15

**Authors:** Monika Angerer, Gerald Pichler, Birgit Angerer, Monika Scarpatetti, Manuel Schabus, Christine Blume

**Affiliations:** Laboratory for Sleep, Cognition and Consciousness Research, Department of Psychology, University of Salzburg, Salzburg, Austria; Centre for Cognitive Neuroscience Salzburg (CCNS), University of Salzburg, Salzburg, Austria; Apallic Care Unit, Albert Schweitzer Hospital, Geriatric Health Care Centres of the City of Graz, Graz, Austria; Private Practice for General Medicine and Neurology, Leibnitz, Austria; Apallic Care Unit, Albert Schweitzer Hospital, Geriatric Health Care Centres of the City of Graz, Graz, Austria; Laboratory for Sleep, Cognition and Consciousness Research, Department of Psychology, University of Salzburg, Salzburg, Austria; Centre for Cognitive Neuroscience Salzburg (CCNS), University of Salzburg, Salzburg, Austria; Centre for Chronobiology, Psychiatric Hospital of the University of Basel, Basel, Switzerland; Transfaculty Research Platform Molecular and Cognitive Neurosciences, University of Basel, Basel, Switzerland

**Keywords:** daylight, light therapy, circadian rhythms, skin temperature, brain injury, disorders of consciousness

## Abstract

**Study Objectives:**

While light therapy has proven effective in re-entraining circadian rhythms, the potential of such an intervention has not been evaluated systematically in post-comatose patients with disorders of consciousness (DOC), who often have strongly altered circadian rhythms.

**Methods:**

We recorded skin temperature over 7–8 days in patients with DOC in each of two conditions: habitual light (HL), and dynamic daylight (DDL) condition. While patients were in a room with usual clinic lighting in the HL condition, they were in an otherwise comparable room with biodynamic lighting (i.e. higher illuminance and dynamic changes in spectral characteristics during the day) in the DDL condition. To detect rhythmicity in the patients’ temperature data, we computed Lomb–Scargle periodograms and analyzed normalized power, and peak period. Furthermore, we computed *inter*daily stability and *intra*daily variability, which provide information about rhythm entrainment and fragmentation.

**Results:**

We analyzed data from 17 patients with DOC (i.e. unresponsive wakefulness syndrome [*n* = 15] and minimally conscious state [*n* = 2]). The period length of the patients’ temperature rhythms was closer to 24 h in the DDL as compared to the HL condition (median median deviation from 24 h: DDL = 0.52 h, HL = 3.62 h). Specifically, in 11/17 (65%) patients the period length was closer to 24 h in the DDL condition. Furthermore, the patients’ rhythm was more pronounced, more stable, and less variable in the DDL condition.

**Conclusions:**

Our results indicate that DDL stimulation entrains and stabilizes circadian rhythms. This highlights the importance of adequate room lighting as an adjunct therapeutic approach for improving circadian rhythms in severely brain-injured patients.

**Trial Registration Information:**

German Clinical Trials Register (DRKS00016041); registration: 18.01.2019; recording start: 04.06.2019 https://www.drks.de/drks_web/navigate.do?navigationId=trial.HTML&TRIAL_ID=DRKS00016041

**Statement of Significance**

In severely brain-injured patients with disorders of consciousness (DOC) circadian (~24 h) rhythms are often strongly altered. While light therapy has shown to be effective in re-entraining circadian rhythms, the potential of such an intervention has not yet been evaluated systematically in patients with DOC. We thus investigated if exposure to biodynamic room lighting, which mimics the spectral composition and changes in illuminance of natural daylight, can support rhythm entrainment. Indeed, we demonstrate that biodynamic room lighting entrains and stabilizes circadian rhythms in patients with DOC after only 1 week of exposure to biodynamic room lighting as compared to standard clinic lighting. This underlines the importance of adequate room lighting in patient rooms as an adjunct therapeutic approach.

## Introduction

Many biological and psychological processes of virtually all living beings follow circadian patterns (i.e. rhythms with a period length of approximately 24 h) and are under tight control of a biological “master clock” that is located in the suprachiasmatic nuclei (SCN) of the hypothalamus [1–3]. The most prominent example for such rhythms is probably the sleep–wake cycle, which is paralleled by circadian changes of other parameters such as body temperature, blood pressure, heart rate (variability), and hormone secretion [4–7].

It is well-known that ambient light is the primary zeitgeber (from German; something that “gives time”/synchronizes) that acts on the SCN [8]. In more detail, especially short-wavelength light (i.e. around 460–480 nm) entrains circadian rhythms. This is primarily through its effects on intrinsically photosensitive ganglion cells in the retina that express the photopigment melanopsin and relay information to the circadian pacemaker in the SCN [9, 10]. While morning light advances the circadian clock, evening and nocturnal light induce phase delays. Based on this knowledge, light stimulation is used as an adjunct or even primary treatment in various fields. Specifically, light stimulation has proven effective in the treatment of circadian rhythm sleep-wake disorders—that is a misalignment between the person’s sleep–wake pattern and the pattern that is desired or considered as the societal norm. These include the advanced and delayed sleep–wake phase disorder, jet lag, shiftwork disorder, sighted non-24-h sleep–wake rhythm disorder and the irregular sleep–wake rhythm disorder (for diagnostic criteria see [11, 12]). Besides this, light also has beneficial effects on mood. This has been suggested to either result from a direct modulation of the availability of neurotransmitters such as serotonin, or from an improved entrainment of circadian rhythms and a resulting reduction in sleep problems, which are common in patients with mental problems. However, the precise mechanisms involved are still a matter of ongoing investigation (for an overview see [13]). Also in healthy individuals, lighting systems that mimic the spectrum of natural daylight were associated with more visual comfort, as well as increased subjective daytime alertness and mood [14].

Besides these findings, it has previously been established that patients with severe brain injury often have strongly altered sleep–wake cycles [15, 16] and circadian rhythms [17–20]. Furthermore, research from our group [21, 22] shows that healthier rhythmicity is associated with a better behavioral repertoire as established during neuropsychological assessment. In more detail, a stronger integrity of patients’ circadian melatonin(-sulfate) and temperature rhythm is related to a richer behavioral repertoire (as measured with the Coma Recovery Scale-Revised; CRS-R [23]). Furthermore, we found stronger circadian components in rhythmicity assessed by (wrist) actimetry in patients with a better clinical state [24].

Thus, given the previously established positive effects of light stimulation in various medical conditions, we sought to investigate if light stimulation can support entrainment of the rhythms of patients with severe brain injury. More specifically, severe brain injury can cause coma and, upon recovery from this state, many patients develop so-called “disorders of consciousness” (DOC). In a simplified approach, consciousness is thought to be constituted of two major components: Wakefulness and awareness [25]. Wakefulness refers to arousal at brain level (i.e. level of consciousness), and awareness to the ability to have a conscious experience of the environment and/or the self (i.e. content of consciousness). In DOC, these two components are altered. Specifically, DOC comprise the unresponsive wakefulness syndrome (UWS) and the minimally conscious state (MCS). While patients with an UWS show some return of arousal (i.e. alternating phases of sleep [closed eyes] and wakefulness [opened eyes]) in the absence of awareness, patients in a MCS show inconsistent but reproducible signs of awareness [26, 27]. When patients regain the ability to functionally communicate and purposefully use objects, their state is classified as emergence from MCS (EMCS) [28]. Furthermore, DOC can be differentiated from coma, which is a state of unarousable unresponsiveness (i.e. no sleep–wake patterns) [29].

Hence, consolidated periods of wakefulness and sleep resulting from well-entrained circadian rhythms seem crucial for adequate arousal levels and thus (conscious) wakefulness. This is why circadian rhythms have received increasing attention in the research of DOC in the last years. However, a question that still remains unanswered is, if there are possibilities to re-entrain such rhythms in patients with DOC, and to improve the patients’ state as a consequence.

Unfortunately, research on light stimulation in patients with DOC is rather scarce. Blume et al. [22] found in an earlier study that two out of eight patients with DOC showed a change of diagnosis from UWS to MCS after stimulation with light enriched in the short-wavelength range (i.e. three times per day for 1 h over the course of 1 week using light therapy glasses with ~2000 photopic lux at eye level). Further investigation of the effects of alterations in daily light exposure in patients with DOC would be of great importance; especially for those patients who are accommodated at long-term care facilities or intensive care units where day and night are often not clearly delineated. Here, photopic illuminance is, compared to light exposure in healthy individuals, usually fairly low during the day and relatively high during the night [30]. This may result in disruptive effects on the circadian system and the sleep–wake cycle [31], which is especially crucial for patients with DOC whose arousal levels anyway strongly fluctuate. Beyond this, it is well-known that an impairment of sleep can have detrimental effects on the immune system and the recovery from illness (for a review see [32]). Eventually, changing room lighting would be a fairly easy to implement and cost-efficient adjunct therapeutic approach that usually comes without any side-effects.

Thus, our aim was to investigate if a dynamic daylight lighting (DDL) characterized by overall increased illuminance and dynamic changes in the spectral distribution (such as in natural daylight) throughout the day can support entrainment of the rhythms of patients with DOC. Specifically, we focused on skin temperature variations, because (i) the integrity of these rhythms has been shown to be predictive for the patients’ behavioral state [21, 22], (ii) the measurement is noninvasive, and (iii) suitable for long-term recordings (i.e. across several days), which is important when investigating circadian rhythmicity.

## Methods

### Experimental design

We recorded data from a total of 18 patients with DOC from a long-term care facility in Austria. Each patient underwent two study conditions (1 week each) in a within-subjects design: (i) habitual light (HL) and (ii) dynamic daylight (DDL) condition. The two conditions differed in light intensity and spectral composition of the patient room lighting (cf. *Habitual light condition*, and *Dynamic daylight condition*). Specifically, besides natural daylight that entered the patients’ room via windows that were oriented to the west, artificial room lighting was manipulated. Each condition comprised seven full days. All patients underwent both conditions, and the order of the conditions was fixed before the start of the recording. We aimed at counterbalancing the order across participants. However, procedures and operations in the clinic precluded a fully counterbalanced design. Specifically, 12 patients were in the HL condition first and 6 patients were in the DDL condition first. If patients were in the DDL condition first, the HL condition was separated by a minimum of one week (*max* = 33 days, *mdn* = 11 days) from the DDL condition to prevent carry-over effects. If patients were in the HL condition first, the DDL condition was separated by a minimum of one day (*max* = 20 days, *mdn* = 2 days) from the HL condition, which enabled the research team to prepare the recordings and move the patients to the room, where the DDL was installed. Importantly, the DDL was the only difference between this room and the HL-room.

During both weeks, temperature, actimetry, and illuminance levels (i.e. photopic lux) were assessed continuously. Electrocardiography (ECG) was recorded at the beginning of each condition for a minimum of 1.5 days (i.e. 36 h continuously; for more information on the ECG recording and analyses please refer to Supplementary file 1). Furthermore, events such as visits or therapies in the patient room were recorded using a tablet (for more details please see [24]). For the current analyses, only the temperature data were of interest. Patients’ behavioral repertoire or level of consciousness was assessed with the CRS-R [23] on 2 days at the end of each condition (i.e. once in the morning and once in the afternoon between days 6 and 8 of each condition; for details cf. *Behavioral assessment and data analysis*).

#### Light measurements.

**Figure 1.**
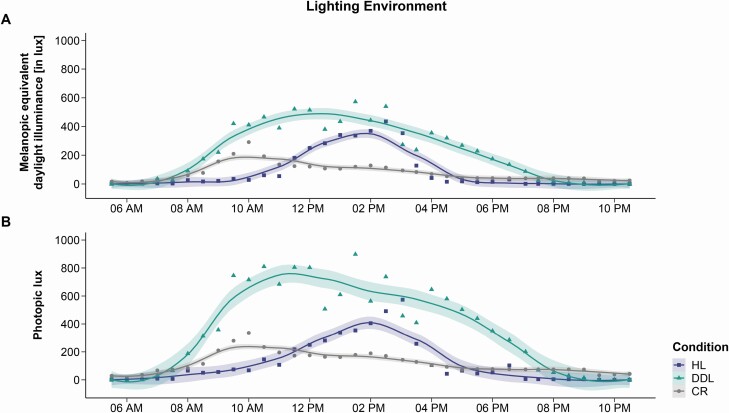
**Lighting environment.** Melanopic equivalent daylight illuminance [M-EDI; in lux] **(A)** and photopic lux **(B)** in the habitual light (HL; purple squares) and dynamic daylight condition (DDL; turquoise triangles), as well as in the common room (CR; grey circles). Measurements were performed at eye level of two patients with one being in the HL and one in the DDL condition, as well as from a pre-specified point (at eye level) in the common room every 30 min from 5:30 am until 10:30 pm at the same day. To visualize the general M-EDI and photopic lux pattern rather than single values that vary with the patients’ head position, we fitted a local regression line into the graph using the “geom_smooth” function of the “ggplot2” package available for R. The span, which controls the amount of smoothing, was set to 0.4; meaning that each of the local regressions used to produce the curve incorporate 40% of the total data points [33].

For a precise description of the light environment, visual (i.e. photopic lux) as well as non-visual components of light (i.e. melanopic equivalent daylight illuminance [M-EDI], which quantifies the primary effects of light on the human circadian clock; cf. Supplementary file 1 for more information) were measured using a spectrometer (GL Spectis 1.0, GL Optic, Weilheim, Germany). Specifically, measurements were performed at eye level of two patients (one being in the HL condition and the other in the DDL condition) every 30 min from 5:30 am until 10:30 pm (i.e. 17-h measurement) on the same day. In parallel, light levels were also measured in the common room at a pre-specified point (that is at the patients’ eye level when sitting in a wheelchair) because patients also spent time there during the recording weeks (cf. Figure 1). However, clinical staff was instructed that the patients should spend as much time as possible in the “study room” (i.e. room with the HL or DDL).

Furthermore, photopic illuminance was monitored by continuous measurements with a light sensor (MotionWatch 8, CamNtech, Cambridge, UK) in both conditions and all patients. The light sensor was fixed to the head of the patients’ bed in the angle of gaze. Clinical staff and relatives were instructed to mount the light sensor on the wheelchair when the patients were mobilized out of their room. These measurements were only included for control purposes of light conditions during data collection (cf. Supplementary file 1: Supplementary Figure S1 to get an impression of these measurements).

#### Habitual light condition.

During the HL condition, patients were in a room with standard clinic lighting (EN 12464-1 lighting standard) that was characterized by an M-EDI range of 0–435 (*mdn* = 27) and a photopic lux range of 4–574 (*mdn* = 67) from 6:30 am (i.e. lights on) until 9:30 pm (i.e. lights off) during the 17-h light measurement (cf. Figure 1). During the night (i.e. 9:30 pm until 6:30 am), light levels were kept at 0 photopic lux except for nocturnal nursing activities, where a brighter light (<50 photopic lux) was needed.

#### Dynamic daylight condition.

During the DDL condition, patients were in a room with a biodynamic patient room light (XAL RECOVER PRO®, XAL Inc., Graz, Austria; complying with the DIN 5035-03 standard for examination rooms and intensive care units). The lighting in the DDL condition was not only characterized by a higher illuminance than the lighting in the HL condition, but also mimicked the natural course of the day through changes in M-EDI and color temperature. Specifically, lighting in the DDL condition was characterized by an M-EDI range of 9–572 (*mdn* = 269) and a photopic lux range of 14–898 (*mdn* = 458) from 6:30 am (i.e. lights on) until 9:30 pm (i.e. lights off) during the 17-h measurement (cf. Figure 1). During the night (i.e. 9:30 pm until 6:30 am), light levels were kept at 0 photopic lux except for nocturnal nursing activities, where a brighter light (<50 photopic lux) was needed. For further information on the installed light program (i.e. change of lumen and color temperature during the day), please refer to Supplementary file 1: Supplementary Figures S2 and S3.

### Behavioral assessment and data analysis

#### Coma recovery scale – revised.

The patients’ behavioral state was assessed behaviorally with the CRS-R [23]. It is composed out of 23 items that are grouped into six subscales reflecting auditory, visual, motor, oromotor, communication and arousal functions. The lowest item on each subscale represents reflexive behavior. The highest item indicates cognitively mediated behavior. Patients are tested in a hierarchical manner, which means that the examiner starts with the highest item of each subscale and moves down the scale until the patient’s response meets the criteria for one item. In both conditions, the assessment was done twice by two trained experts. For our analyses, we used those CRS-R assessments of each condition where the patients showed the highest behavioral reactivity (i.e. the best diagnosis or highest sum score) as this is thought to best represent the true state of the patient. The highest CRS-R score and diagnosis of each patient is shown separately for HL and DDL condition in Table 1.

**Table 1.** Demographic information and highest CRS-R score/diagnosis separately for each condition

Patient ID	Recording period (quarter)	First condition	Age	Gender	Etiology	Time since injury (months)	Pupillary light reflex	Diagnosis HL	CRS-R sum score HL	Diagnosis DDL	CRS-R sum score DDL
P1	2	HL	63.6	M	NTBI	8.8	Yes	UWS	3	UWS	4
P2	2	DDL	77.0	M	NTBI	15.7	Yes	UWS	3	UWS	3
P3	3	HL	61.6	F	NTBI	20.6	Yes	UWS	6	UWS	6
P4	3	HL	34.7	M	NTBI	14.6	Yes	UWS	4	UWS	4
P5	3	HL	71.2	M	NTBI	20.7	Yes	EMCS	21	EMCS	22
P6	3	DDL	55.2	F	NTBI	23.8	Yes	UWS	4	UWS	4
P7	3	HL	58.8	M	NTBI	25.8	Yes	UWS	4	UWS	4
P8	4	HL	80.3	M	TBI	21.9	Yes	MCS	9	-	-
P9	4	DDL	22.3	F	NTBI	48.3	Yes	-	-	UWS	4
P10	4	HL	47.6	M	TBI	7.7	Yes	UWS	5	UWS	5
P11	4–1	DDL	69.0	F	NTBI	10.3	Yes	UWS	5	UWS	6
P12	4	DDL	55.4	M	NTBI	12.5	Yes	UWS	5	UWS	5
P13	4	HL	70.0	F	NTBI	25.9	No	MCS	8	UWS	6
P14	4	HL	76.3	M	TBI	3.5	Yes	UWS	4	MCS	9
P15	4	DDL	65.0	M	NTBI	6.0	Yes	-	-	-	-
P16	4	HL	69.9	M	NTBI	3.4	Yes	UWS	2	-	-
P17	1	HL	74.4	M	NTBI	9.1	Yes	UWS	4	UWS	5
P18	1	HL	70.6	F	TBI	4.7	Yes	UWS	4	UWS	4

*Recording period (quarter)* Period in which the recording took place, 1: January–March, 2: April–June, 3: July–September, 4: October–December; *First Condition* Condition that was first, HL: habitual light condition first, DDL: dynamic daylight condition first; *M* male; *F* female; *NTBI* non-traumatic brain injury; *TBI* traumatic brain injury; *UWS* unresponsive wakefulness syndrome; *MCS* minimally conscious state; *EMCS* emergence from MCS; *CRS-R* Coma Recovery Scale-Revised *(cf. Behavioral assessment and data analysis)*. Please note that we could not obtain valid CRS-R assessments in four patients (P8, P9, P15, P16) in at least one of the two conditions because not all subscales could be evaluated (e.g. due to eyes being closed and it being impossible to induce eye-opening even when physically stimulating the patients).

#### Skin temperature.

We recorded variations in skin temperature with a sampling rate of 1/300 Hz (i.e. one sampling point every five minutes) using external skin sensors (iButton DS1922L, Maxim Integrated Products Inc., San Jose, CA). Two sensors were affixed in an infraclavicular position on the right and left body side (=proximal location relative to the body center), and two on the left and right ankle (=distal location relative to the body center) using medical tape. Additionally, one sensor was placed in the room close to the patient to measure room temperature. Temperature sensors recorded continuously during both conditions (i.e. seven full days during HL and DDL condition) and were only taken off if the patients were showered or bathed. In three subjects we could only use temperature data from the first 6 days of the DDL condition due to wrong placement of the distal sensors on day 7 (P17), or terminating the DDL stimulation on day 6 already because of a communication problem (P4, P13).

Artefact correction and analysis of temperature data was done in R version 3.6.1 [34] according to a routine used in previous publications from our group [21, 22]. First, values deviating more than 2.5 times the interquartile range were removed (i.e. capturing times when the sensors were taken off during showering and bathing). In total, we removed 0.4% (*mdn*) of the data points in the HL condition (*min* = 0%, *max* = 6.3%) and 0.15% (*mdn*) of the data points in the DDL condition (*min* = 0%, *max* = 3.2%). Second, the distal-proximal gradient (DPG), which has been shown to serve as a proxy for core body temperature [35], was computed by subtracting the mean of the two distal sensors from the mean of the two proximal sensors at each recorded time point. Afterwards the grand average of the DPG was subtracted from every DPG value in a pointwise manner as suggested by van Dongen et al. [36]. Note that in three patients only one distal sensor could be used for the DPG computation in one of the two conditions (i.e. twice because of a technical error [P2—HL condition, P12—HL condition] and once because the second distal sensor got lost [P17—DDL condition]), which may have resulted in a slightly noisier signal.

#### Lomb–Scargle periodograms.

To detect rhythmicity in our data, we computed Lomb–Scargle periodograms [37, 38] based on the DPG using the “lomb” package available for R [39] as in previous publications [21, 22, 24]. In each patient, we were interested in two parameters: (i) normalized power, and (ii) peak period. For the calculation of normalized power, a sine wave is fitted to the data. Normalized Power is maximal where the sum of squares of the fitted sine wave to the data are minimal. For calculation of the period length of each patient’s temperature rhythm, we looked for significant peaks in the normalized power of the periodogram and extracted the period length of the strongest peak within 24 ± 12 h. We set the oversampling factor to 100 and the significance level to *α* = .001. The individual patients’ results are displayed in Supplementary File 1: Supplementary Tables S1 and S2. For our analyses, we computed the deviation of the patients’ period length from 24 h (i.e. as circadian rhythms should be entrained to a 24 h cycle in a natural setting, which is close to the intrinsic period of the human circadian pacemaker that is on average 24.18 h [40]). For further information on the analyses please refer to the supplementary material of Blume et al. [21].

#### Interdailiy stability and intradaily variability.

*Inter*daily stability (IS) and *intra*daily variability (IV) are nonparametric measures [41], which were originally suggested for the analysis of actigraphy data, and whose calculation is implemented in the R package “nparACT” [42]. In more detail, IS quantifies how well a rhythm, i.e. the patients’ temperature rhythm, is entrained to a 24 h zeitgeber (i.e. light–dark cycle). It ranges between 0 for Gaussian noise and 1 for perfect IS. IV describes how fragmented the rhythm is. It converges to 0 for a perfect sine wave, approaches 2 for Gaussian noise, and may be even higher when a definite ultradian component with a period length of about 2 h is present.

As IS does not allow for missing values (i.e. in our case due to the artefact correction mentioned above), the first half of the missing values was imputed by the last valid temperature value before the first missing value. The second half of the missing values (plus one value—in case of an uneven amount of missing values) was imputed by the first valid temperature value after the last missing value. A single missing value was replaced by the mean of the last value before and the first value after the missing value. Additionally, we normalized the DPG data between 0 and 1 to get rid of negative values. This procedure left the rhythmicity of the data unaffected. For individual patients’ results please refer to Supplementary file 1: Supplementary Tables S1 and S2.

### Statistical analyses

Statistical analyses were done in R version 3.6.1 [34]. As not every variable was normally distributed (i.e. Shapiro–Wilk test for normality: *p* < .001; cf. Supplementary file 1: Supplementary Table S3), we used an advanced nonparametrical statistical approach. The significance level was set to *α* = .05 (one-sided) for all our analyses. As suggested by Wasserstein et al. [43], we interpreted the overall pattern rather than focusing on individual *p*-values. Therefore, we also interpreted *p*-values .05 < *p* ≤ .1 if they were in line with the overall pattern.

For the analyses of differences in circadian skin temperature parameters (i.e. deviation of the peak period from a 24-h rhythm, normalized power, IS, IV) between the HL and DDL conditions, we used advanced nonparametric analyses of longitudinal data as implemented in the “nparLD” package available for R [44], a method which is suitable for our data as it is free of distributional assumptions. We report the ANOVA-type statistic (ATS) along with the Relative Treatment Effect (RTE). The RTE can range between 0 and 1, and describes the probability of a randomly chosen value from the respective group (i.e. HL or DDL) being larger than a randomly chosen observation from the whole dataset (i.e. HL and DDL together).

For correlation analyses of time since injury, age, CRS-R scores, and temperature data, we report Kendall’s Tau. To correct for multiple tests, *p*-values of all correlation analyses were adjusted using the method of Benjamini and Hochberg [45] as implemented in the “p.adjust” function in R.

## Results

### Patient demographics

In the HL condition, 13 patients were diagnosed with UWS, two were in an MCS, one was in an EMCS and two could not be diagnosed (P9, P15). In the DDL condition, 13 patients were diagnosed with UWS, one was in an MCS, one in an EMCS and three could not be diagnosed (P8, P15, P16). We could not obtain valid CRS-R assessments in four patients (P8, P9, P15, P16) in at least one of the two conditions, because not all subscales could be evaluated (i.e. due to eyes being closed and it being impossible to induce eye-opening even when physically stimulating the patients; cf. Supplementary file 1 for more information). In patients P13 and P14, the diagnosis differed between the HL and DDL conditions; in the other patients the diagnosis did not change. As we were interested in the effects of light stimulation on circadian rhythms in patients with severe brain injury, we excluded one patient who was at the transition to recover from DOC (i.e. EMCS; P5) from the following analyses. Further inclusion criteria were: male or female sex, and an age between 18 and 80 years. Exclusion criteria were: paroxysmal sympathetic hyperactivity, and strong vegetative reactions (dysautonomia) and/or motor restlessness as a result of the test protocol. These criteria are also documented in the pre-registration of the study (cf. *German Clinical Trials Register; DRKS00016041*). Thus, 17 patients (six women) aged 20.3–80.3 years (*mdn* = 65) were included in the study sample. Please note that sedative medication was comparable between conditions and none of the patients showed signs of acute infections during the study period. For more details on the study sample, please see Table 1.

### Habitual light vs. dynamic daylight condition

**Figure 2.**
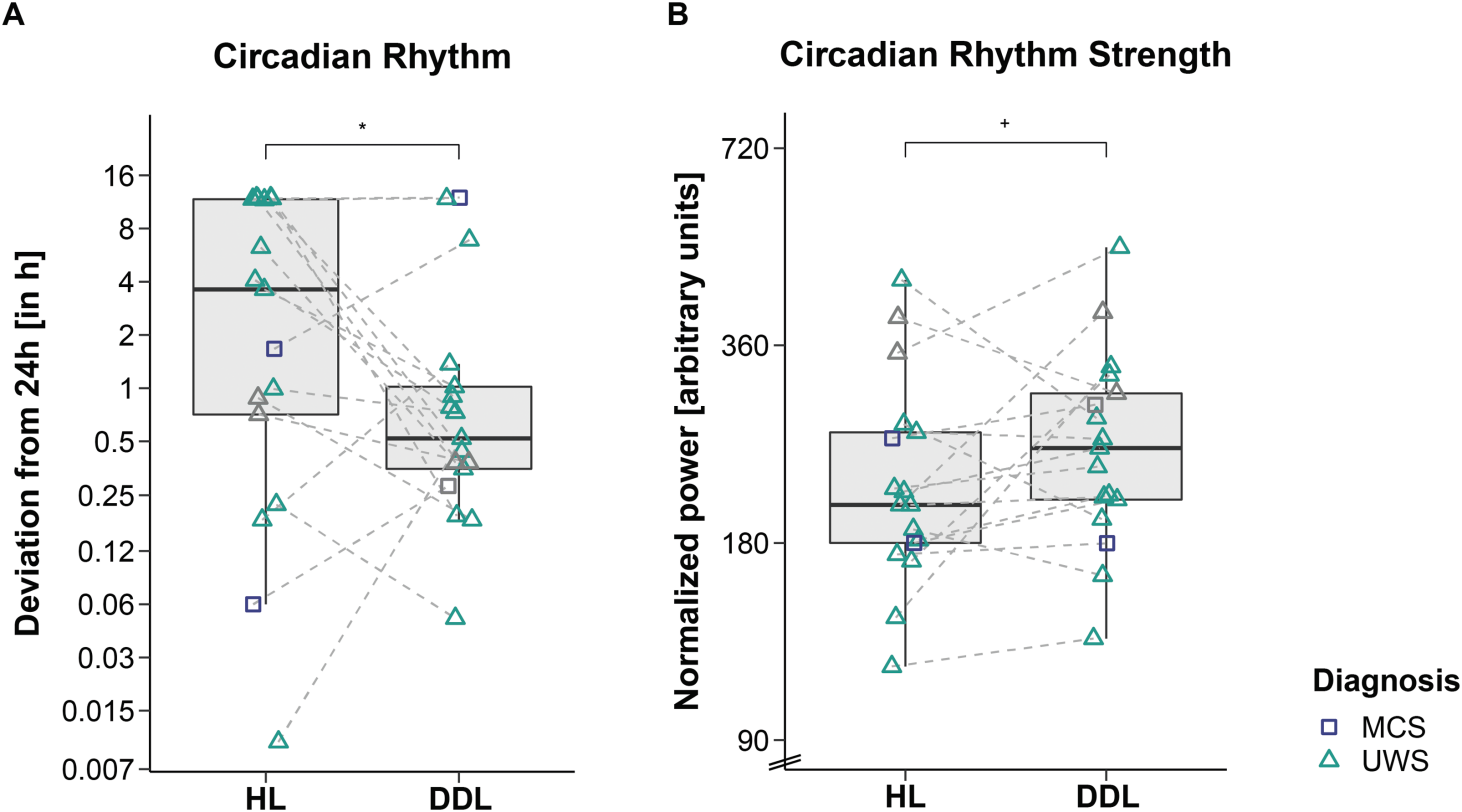
**Circadian rhythmicity (A) and rhythm strength (B) in habitual light (HL) vs. dynamic daylight (DDL) condition.** (A) Deviation of the patients’ global peak period from 24 h. The patients’ temperature rhythms were significantly better entrained to a 24-h rhythm in the DDL condition (i.e. they deviated less from 24 h). (B) Normalized power of the patients’ global peaks. The normalized power was by trend higher in the DDL condition. For better illustration, the data are shown on a log-scale axis. Horizontal lines represent the medians, boxes the interquartile range (IQR; distance between the 1st [Q1] and 3rd quartile [Q3]), and whiskers extend at most to Q1−1.5*IQR (lower whisker) and Q3 + 1.5*IQR (upper whisker). Asterisks indicate significance: *p < .05, ^+^*p* ≤ .1. Abbreviations: *MCS* minimally conscious state, *UWS* unresponsive wakefulness syndrome. The symbols in grey show patients from whom we could not obtain valid CRS-R assessments during one of the two conditions (P8, P9, P16). In this case, we generalized the diagnoses from one condition to the other for visualization purposes only (statistical evaluation of diagnoses was not included as a factor). In one patient (P15), no valid CRS-R assessment could be obtained in any of the conditions. Here, we used the latest diagnosis from the clinic (i.e. UWS), which was obtained one month before the start of the recording.

**Figure 3.**
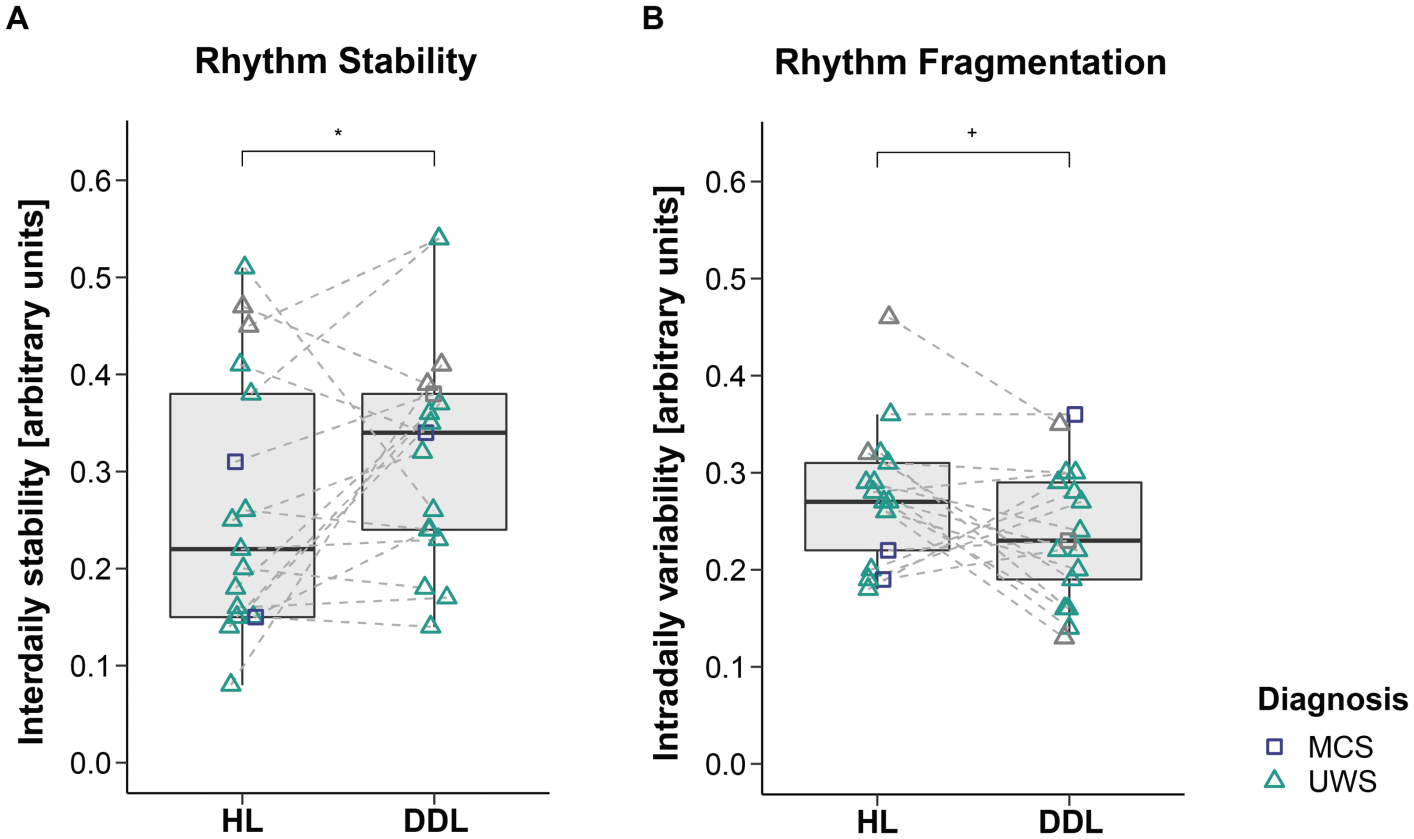
**Rhythm stability (A) and fragmentation (B) in habitual light (HL) vs. dynamic daylight (DDL) condition.** (A) *Inter*daily stability (IS). The IS was significantly higher in the DDL condition (IS approaches 0 for Gaussian noise and converges to 1 for perfect IS). (B) *Intra*daily variability (IV). The IV was by trend lower in the DDL condition (IV converges to 0 for a perfect sine wave [i.e. no IV] and approaches 2 for Gaussian noise.). Horizontal lines represent the medians, boxes the interquartile range (IQR; distance between the 1st [Q1] and 3rd quartile [Q3]), and whiskers extend at most to Q1−1.5*IQR (lower whisker) and Q3 + 1.5*IQR (upper whisker). Asterisks indicate significance: *p < .05, ^+^*p* ≤ .1. *MCS* minimally conscious state, *UWS* unresponsive wakefulness syndrome. The symbols in grey show patients from whom we could not obtain valid CRS-R assessments during one of the two conditions (P8, P9, P16). In this case, we generalized the diagnoses from one condition to the other for visualization purposes only (statistical evaluation of diagnoses was not included as a factor). In one patient (P15), no valid CRS-R assessment could be obtained in any of the conditions. Here, we used the latest diagnosis from the clinic (i.e. UWS), which was obtained one month before the start of the recording.

Comparisons between HL and DDL temperature data revealed that the period length of the patients’ temperature rhythm was closer to 24 h in the DDL condition (*F*_*ATS*_(1) = 3.18, *p* = .037, *RTE*_*HL*_ = .58*, RTE*_*DDL*_ = .42; median deviation from 24 h: HL condition = 3.62 h, DDL condition = 0.52 h; cf. Figure 2A). The circadian rhythm (i.e. normalized power) was by trend stronger in the DDL condition as compared to the HL condition (*F*_*ATS*_(1) = 2.28, *p* = .066, *RTE*_*HL*_ = .44*, RTE*_*DDL*_ = .56; cf. Figure 2B). The *inter*daily stability was higher in the DDL condition than in the HL condition (*F*_*ATS*_(1) = 3.29, *p* = .035, *RTE*_*HL*_ = .43*, RTE*_*DDL*_ = .57; cf. Figure 3A). The *intra*daily variability was by trend lower in the DDL condition as compared to the HL condition (*F*_*ATS*_(1) = 1.90, *p* = .084, *RTE*_*HL*_ = .56*, RTE*_*DDL*_ = .44; cf. Figure 3B).

## Discussion

We here find that biodynamic room lighting mimicking daylight regarding changes in photopic illuminance, melanopic EDI, and color temperature can support adequate entrainment and stabilization of circadian rhythms in immobile patients suffering from severe brain injury. More specifically, dynamic daylight shifted the period length of patients’ temperature rhythms closer to 24 h, and amplified the strength of these rhythms. In more detail, in 11/17 (65%) patients the period length was closer to 24 h in the DDL condition (cf. Supplementary file 1: Supplementary Tables S1 and S2). Furthermore, the stability of circadian variations *across* days increased while variability *within* the day decreased. This means that patients’ rhythm follows a similar pattern over several days (i.e. higher *interdaily* stability—indicating a better entrainment to a 24-h rhythm) and is less fragmented within a day (i.e. lower *intradaily* variability—meaning that the patients temperature variation throughout a day is closer to a sine wave). Thus, more clearly delineated day and night periods in the DDL condition may lead to better consolidated phases of wakefulness and sleep, and in consequence to more stable rhythms [13]. Hence, dynamic daylight exposure may be an easy way to implement a relatively low-cost adjunct therapeutic approach in immobile patient populations.

Additionally, when looking at correlations between the circadian parameters mentioned above, we found stronger effect sizes for intercorrelations between those parameters in the DDL condition as compared to the HL condition, which also points toward an entraining effect of the DDL stimulation. In more detail, patients, whose temperature rhythms deviated less from 24 h, showed a more stable (i.e. higher IS) and a more pronounced circadian rhythm (i.e. higher normalized power). Also, patients with a more pronounced circadian rhythm (i.e. higher normalized power) had a more stable rhythm over days (i.e. higher IS). In contrast to previous studies from our lab [21, 22], we did not find direct associations between circadian temperature parameters and patients’ behavioral repertoire (cf. Supplementary file 1: Supplementary Figure S5). This could be due to the fact that patients from our current study were, on average, in a more acute state than patients included in previous studies, which also may have contributed to the little interindividual variability in the CRS-R scores in the present study.

In additional analyses, we were looking at the patients’ behavioral level and found that patients showed by trend a richer behavioral repertoire at assessments with the CRS-R during the DDL condition (cf. Supplementary file 1: Supplementary Figure S4). However, no systematic change in clinical diagnosis (i.e. UWS, MCS) could be observed. Thus, although already one week of light stimulation had a positive impact on the integrity of the patients’ circadian temperature rhythms, it did not lead to a change in diagnosis. Possibly, a longer-lasting DDL exposure, especially in such chronic conditions, would be necessary for such an effect to be observed. Thus, further studies are needed that investigate if light stimulation over a longer period of time can result in an improvement of the patients’ consciousness levels.

Furthermore, our study sample did not allow for comparisons between diagnoses or etiologies. Future studies should therefore investigate whether some patient characteristics are predictive for patients being responsive to the light intervention. Specifically, various therapeutically approaches are more effective in patients with MCS as compared to patients with UWS. Our study sample only comprised two patients with MCS, but 15 patients with UWS. This might also explain why we did not find an effect of DDL exposure on patients’ diagnosis. Additionally, it should be examined if lighting conditions that are able to entrain temperature rhythms of patients with severe brain injuries also stabilize the sleep–wake cycle; especially, because we know that body temperature is involved in regulating the sleep–wake cycle [46], which is strongly fragmented in most patients with DOC [15]. The latter is particularly critical because sleep is important for the immune system, recovery from illness and psychological well-being [32]; aspects that are all relevant for patients with DOC who have to recover from the brain injury.

Another possible limitation of the present study is that no in-depth assessments of the patients’ optical system (e.g. magnetic resonance imaging scans) were available. All but one patient showed a pupillary light reflex (cf. Table 1); however, the absence of a pupillary light reflex can have manifold reasons and is not to be equated with the absence of any response to light and thus its circadian effects. Future studies should evaluate the integrity of the optical system and include a quantitative assessment of the pupillary light reflex.

As mentioned above, we were not able to fully counterbalance our sample due to procedures in the clinic, which resulted in more patients having undergone the HL condition first. Importantly though, follow-up analyses showed that this did not influence the results (cf. Supplementary file 1).

To summarize, we find that dynamic daylight stimulation entrains and stabilizes the rhythm of patients with severe brain injuries. Our findings highlight the beneficial effects of adequate room lighting and thus its relevance especially in intensive care units and long-term care facilities. In particular because we know from studies in healthy individuals that nighttime exposure to typical room light (i.e. 90–180 lux) can already have an alerting effect [47]. Therefore, apart from light that is needed during nursing activities, light levels in the patient room should be kept as low as possible during the night. Furthermore, our results underline the importance of more naturalistic lighting systems in patient rooms, which are not only characterized by a higher photopic illuminance during the day but also mimic the natural variations in the spectral composition of daylight, thereby taking the importance of nonvisual (i.e. circadian) effects of light into account.

## Supplementary Material

zsac065_suppl_Supplementary_Material

## Data Availability

The data that support the findings of this study are available from the corresponding author upon reasonable request.

## Preprint Repositories

A preprint of this manuscript was previously published on Open Science Framework (OSF). doi: 10.31219/osf.io/vk2d6.
