# Correction to: From dawn to dusk—mimicking natural daylight exposure improves circadian rhythm entrainment in patients with severe brain injury

**DOI:** 10.1093/sleep/zsac186

**Published:** 2022-08-08

This is a correction to: Monika Angerer, Gerald Pichler, Birgit Angerer, Monika Scarpatetti, Manuel Schabus, Christine Blume, From dawn to dusk—mimicking natural daylight exposure improves circadian rhythm entrainment in patients with severe brain injury, *Sleep*, Volume 45, Issue 7, July 2022, zsac065, https://doi.org/10.1093/sleep/zsac065

In the originally published version of this manuscript an earlier supplementary material file was published. In the updated file (now corrected online) the changes made were:

The authors have reported the one-sided test instead of the two-sided version in two cases, where p-values changed from .966 to .483 and .665 to .333. This did not change the results and only concerned numerical changes.

Further, the authors corrected the reported degrees of freedom for the correlations.

Lastly, the authors changed the way degrees of freedom were reported for the results from R’s nparLD package because they had learned that with this statistical approach they should report the degrees of freedom with decimals instead of the ANOVA degrees of freedom.

The publisher apologizes for this error, which has now been corrected.

