# Supplementary material for: From dawn to dusk—mimicking natural daylight exposure improves circadian rhythm entrainment in patients with severe brain injury
Source: Sleep. 2022 Mar 15;45(7):zsac065. doi: 10.1093/sleep/zsac065 (PMC9272242; doi:10.1093/sleep/zsac065)
Supplement: zsac065_suppl_Supplementary_Material [file zsac065_suppl_supplementary_material.pdf]

From dawn to dusk—mimicking natural daylight exposure improves circadian rhythm entrainment in patients with severe brain injury

SUPPLEMENTARY MATERIAL

Monika Angerer[1,2], Gerald Pichler[3], Birgit Angerer[4], Monika Scarpatetti[3], Manuel Schabus[1,2*], Christine Blume[5,6*]

*these authors contributed equally

Affiliations:

[1] Laboratory for Sleep, Cognition and Consciousness Research; Department of Psychology; University of Salzburg; Salzburg, Austria

[2] Centre for Cognitive Neuroscience Salzburg (CCNS); University of Salzburg; Salzburg, Austria

[3] Apallic Care Unit; Albert Schweitzer Hospital; Geriatric Health Care Centres of the City of Graz; Graz, Austria

[4] Private Practice for General Medicine and Neurology; Leibnitz, Austria

[5] Centre for Chronobiology; Psychiatric Hospital of the University of Basel; Basel, Switzerland

[6] Transfaculty Research Platform Molecular and Cognitive Neurosciences; University of Basel; Basel, Switzerland

Corresponding Authors:

Monika Angerer
Email: monika.angerer@plus.ac.at

Christine Blume
Email: christine.blume@unibas.ch

Methods

Additional Information on Missing CRS-R Data

We could not obtain valid CRS-R assessments in four patients (P8, P9, P15, P16) in at least one of the two conditions, because not all subscales could be evaluated (i.e. due to eyes being closed and it being impossible to induce eye-opening even when physically stimulating the patients). As the patients showed eye opening on other assessments and occasions, it can be assumed that fluctuations in eye opening are related to fluctuations in vigilance rather than a stable mental state (or consciousness level). In such a case (i.e. when the patient is asleep), it is invalid to assess the patients' visual function. More specifically, scoring patients' performance on the visual subscale with 0 (i.e. when the patients show no reactions to any item of the subscale) after manually opening the patients' eyelids, would not validly reflect the patients' state as visual perception (including visual startle) requires at least wake-like sensory processing, even in UWS.

Melanopic Equivalent Daylight Illuminance

Melanopic equivalent daylight illuminance (M-EDI) quantifies the effects of light on the human circadian rhythm. It can be derived from multiplying the photopic illuminance (in lux) with the melanopic/photopic ratio (M/P ratio) from the spectrum of incident light. In more detail, the M/P ratio is the ratio of the melanopic response from melanopsin-containing intrinsically photosensitive retinal ganglion cells in the eye (i.e. that are involved in controlling circadian rhythms) to the visual response. This measure allows comparing light settings to natural daylight and gives a more complete description of light settings in studies. The latter is important for comparability and reproducibility of study results and lighting conditions [1].

Photopic Illuminance

Photopic illuminance was monitored with continuous measurements with a light sensor (MotionWatch 8, CamNtech, Cambridge, United Kingdom) in both conditions and all patients. The light sensor was fixed to the head of the patient's bed. Clinical staff and relatives were instructed to mount the light sensor on the wheelchair when the patients were mobilized out of their room. These measurements were only included for control purposes of light conditions during data collection (cf. *Figure S1* to get an impression of these measurements).

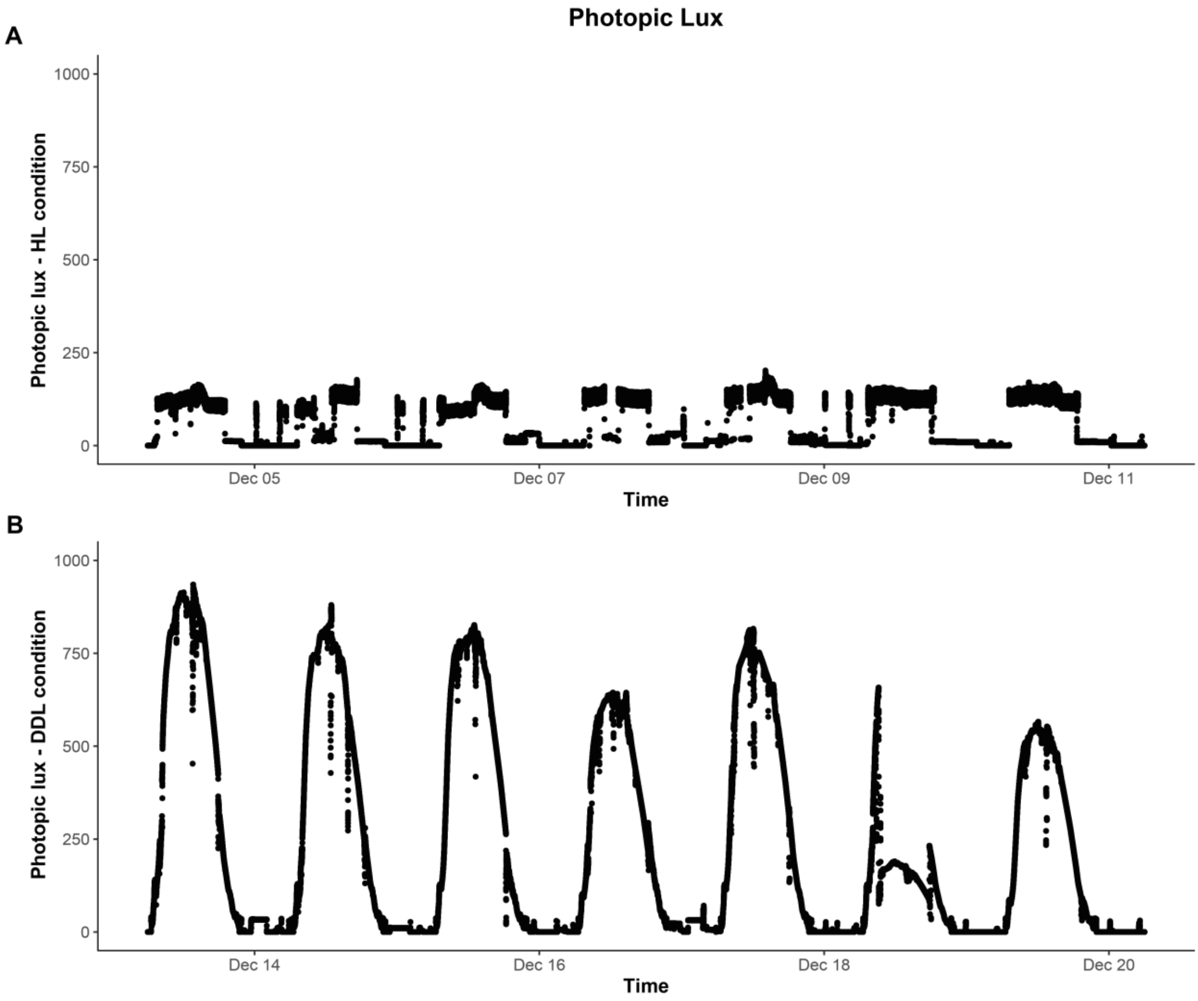

***Figure S1.* Exemplary data (patient P16) of continuous photopic lux measurements over the course of one week in the habitual [HL] (A), and dynamic daylight [DDL] condition (B).** Overall, photopic lux levels were up to four times higher in the DDL condition as compared to the HL condition. While the DDL condition was characterized by a continuous in- and decrease of photopic lux levels, this was not the case in the HL condition because here, light was not manipulated in a circadian manner. The abrupt decrease in photopic lux in the DDL condition on the 18th of December was probably caused by a shift in the position of the light sensor with the sensor being directed against the wall.

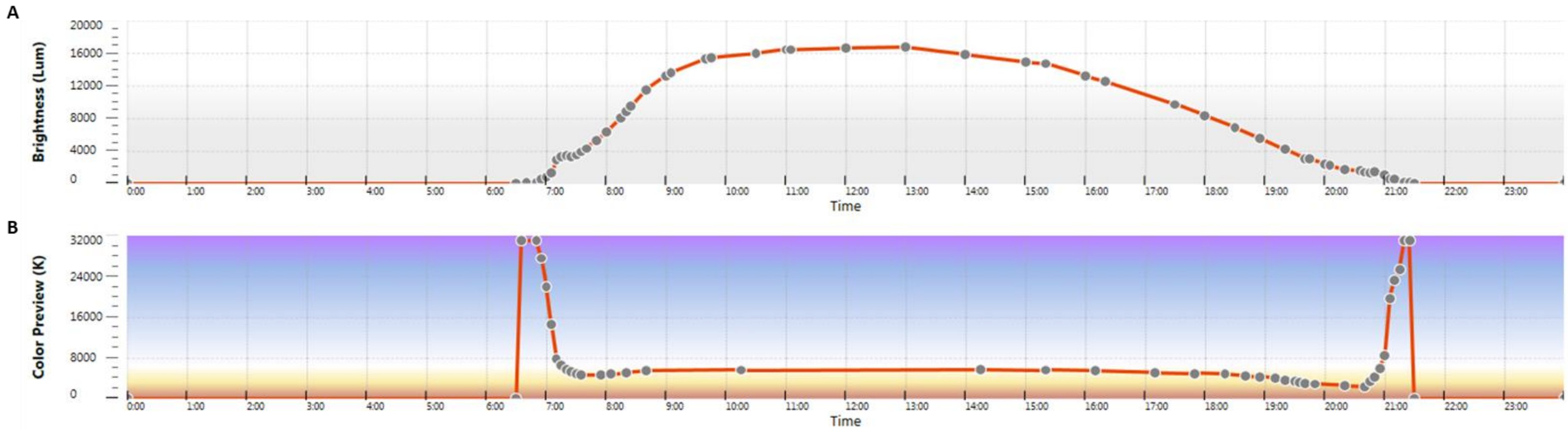

***Figure S2.* Changes in brightness [in lumen] (A) and color temperature [in kelvin] (B) of the biodynamic patient room light in the dynamic daylight (DDL) condition.**

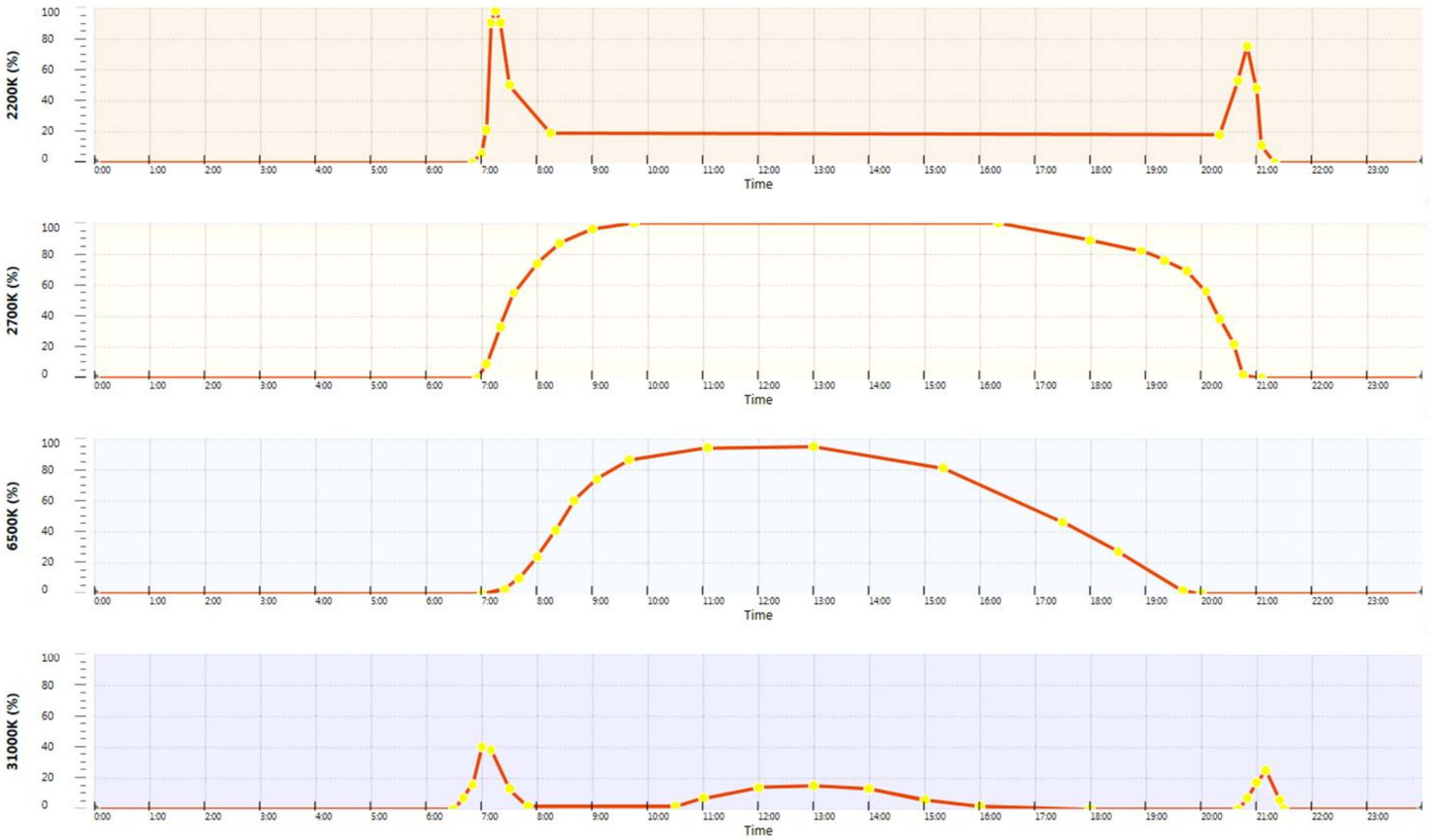

Figure S3. **Intensity curves of the four color temperatures, from which the intensity and color temperature curves in the dynamic daylight (DDL) condition** (cf. *Figure S2*) **result.**

Results

Individual Patient Results

Table S1.
Circadian rhythm indices from the habitual light condition.

Patient ID	Interdaily Stability (IS)	Intradaily Variability (IV)	Peak Period	Normalized Power
P1	0.38	0.20	23.78	265.40
P2	0.15	0.32	19.92	217.90
P3	0.18	0.29	35.80	168.84
P4	0.25	0.27	23.99	181.79
P5	0.35	0.31	23.90	254.72
P6	0.16	0.28	24.99	188.72
P7	0.51	0.31	23.82	453.56
P8	0.31	0.22	24.06	259.94
P9	0.45	0.32	24.88	350.83
P10	0.26	0.27	20.38	273.21
P11	0.15	0.26	35.96	138.39
P12	0.22	0.29	12.37	215.50
P13	0.15	0.19	22.33	179.92
P14	0.41	0.36	12.19	172.73
P15	0.47	0.46	23.29	397.61
P16	0.08	0.26	30.26	205.63
P17	0.14	0.18	35.96	205.55
P18	0.20	0.19	12.28	116.50

Peak period refers to the strongest peak (i.e. highest normalized power) within 24 ± 12 h. Markings in grey indicate period lengths that deviate > 1 h from 24 h. This was the case in 10/18 (55.6 %) patients.

Table S2.
Circadian rhythm indices from the dynamic daylight condition.

Patient ID	Interdaily Stability (IS)	Intradaily Variability (IV)	Peak Period	Normalized Power
P1	0.54	0.28	23.95*	259.42
P2	0.24	0.16	23.22*	235.16
P3	0.36	0.24	24.44*	324.64
P4	0.32	0.16	24.52	213.02
P5	0.18	0.28	26.91	207.60
P6	0.17	0.3	24.73*	160.41
P7	0.26	0.3	25.37	279.17
P8	0.38	0.23	23.72	292.47

P9	0.54	0.19	23.82*	508.65
P10	0.24	0.22	25.02*	195.60
P11	0.37	0.2	23.65*	334.40
P12	0.23	0.14	23.10*	251.11
P13	0.14	0.22	17.11	209.47
P14	0.34	0.36	12.05	179.72
P15	0.39	0.35	23.62*	304.44
P16	0.41	0.13	23.62*	404.78
P17	0.35	0.29	24.19*	211.49
P18	0.18	0.27	12.19	128.51

Peak period refers to the strongest peak (i.e. highest normalized power) within 24 ± 12 h. Markings in grey indicate period lengths that deviate > 1 h from 24 h. This was the case in 6/18 (33.3 %) patients. Asterisks indicate patients whose period length was closer to 24 h in the DDL condition compared to the HL condition.

Normality Test Results

Table S3.
Shapiro-Wilk tests for normality separately for variables of the habitual light (HL) and dynamic daylight (DDL) condition. *P*-values for age and time since injury refer to both HL and DDL condition. Significant *p*-values (i.e. <.05) indicating that the data is not normal distributed are marked in grey.

Variable		Shapiro-Wilk tests (*p*-values) - HL	Shapiro-Wilk tests (*p*-values) - DDL
Deviation from 24h		< .001	< .001
Normalized power		.038	.242
Interdaily stability		.075	.413
Intradaily variability		.155	.674
CRS-R sum score		.034	.011
CRS-R auditory subscale score		< .001	.003
CRS-R visual subscale score		< .001	< .001
CRS-R motor subscale score		< .001	< .001
CRS-R oroverbal subscale score		< .001	< .001
CRS-R communication subscale score		-	-
CRS-R arousal subscale score		< .001	< .001
Age	.042		
Time since injury	.017		

Due to little variability in the CRS-R communication subscale scores, no Shapiro-Wilk tests can be reported.

CRS-R Scores – HL vs. DDL Condition

When comparing data between lighting conditions only patients with valid CRS-R scores in both conditions were included. More specifically, as we could not obtain valid CRS-R assessments in four patients [P8, P9, P15, P16] in at least one of the two conditions, we decided for a case-wise deletion. Thus, 13/17 patients were included in the following analyses.

Comparisons of CRS-R scores between HL and DDL condition showed that the patients' CRS-R sum score was significantly higher in the DDL condition as compared to the HL condition (F_{ATS}(1)=3.26, p=.036, RTE_{HL}=.45, RTE_{DDL}=.55; cf. *Figure S4A*). The analyses also yielded significance when comparing auditory subscale scores between conditions with higher scores in the DDL condition (F_{ATS}(1)=5.33, p=.01, RTE_{HL}=.43, RTE_{DDL}=.57). However, due to little variability in the scores, we deliberately refrain from interpreting this result. No differences between conditions could be found when looking at the visual (F_{ATS}(1)=0.002, p=.483, RTE_{HL}=.5, RTE_{DDL}=.5) and arousal subscale scores (F_{ATS}(1)=0.19, p=.333, RTE_{HL}=.48, RTE_{DDL}=.52). Due to little variability in the communication, oroverbal, and motor subscale scores, no statistics can be reported.

Additionally, we ran the analysis on the comparison of CRS-R sum scores between conditions again without the inclusion of patient P14. More specifically, this patient was in the HL condition first and showed an improvement of five points in the CRS-R sum score from the HL to the DDL condition. As P14 had a traumatic brain injury, it is possible that this is an effect of spontaneous recovery rather than the light stimulation. After exclusion of P14, results still show that patients' CRS-R sum scores were by trend higher in the DDL condition as compared to the HL condition (F_{ATS}(1)=2.34, p=.063, RTE_{HL}=.47, RTE_{DDL}=.53; *Figure S4B*). Thus, the observed effect does not seem to be entirely driven by P14.

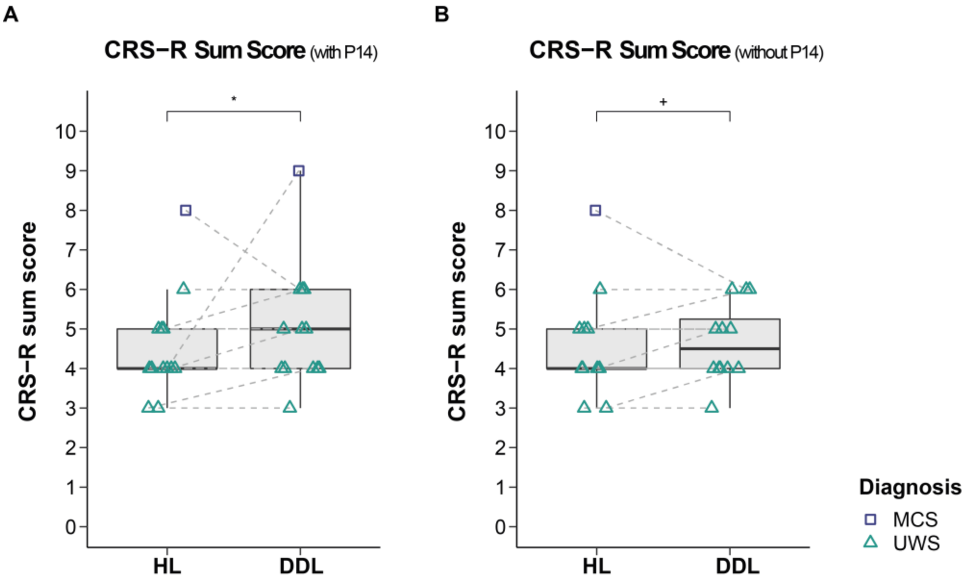

Figure S4. **CRS-R sum score in the habitual light (HL) vs. dynamic daylight (DDL) condition with (A) and without (B) inclusion of patient P14.** (A) Patients showed a significantly higher CRS-R sum score in the DDL condition. (B) Patients showed by trend a higher CRS-R sum score in the DDL condition. Horizontal lines represent the medians, boxes the interquartile range (IQR; distance between the 1st [Q1] and 3rd quartile [Q3]), and whiskers extend at most to Q1−1.5*IQR (lower whisker) and Q3+1.5*IQR (upper whisker). Asterisk indicates significance: $^{*}p < .05$, $^{+}p \leq .1$. Abbreviations: *MCS* minimally conscious state, *UWS* unresponsive wakefulness syndrome. Note that the analyses only included patients with valid CRS-R scores for both conditions (A: *n*=13, B: *n*=12).

Correlation between Age, Time Since Injury, CRS-R Scores and Temperature Data

Our results revealed positive correlations between the CRS-R sum score and CRS-R visual subscale score in both conditions (HL: $r_\tau(13)=0.78$, $p=.004$; DDL: $r_\tau(12)=0.67$, $p=.026$). Furthermore, the CRS-R sum score correlated positively with the CRS-R oroverbal subscale score in the DDL condition ($r_\tau(12)=0.66$, $p=.034$).

Additionally, less deviation of the patients' peak from 24 h was associated with (i) a higher normalized power of the patients' rhythm (HL: $r_\tau(15)=-0.45$, $p=.054$; DDL: $r_\tau(15)=-0.45.$, $p=.039$) and (ii) a higher interdaily stability [IS] (HL: $r_\tau(15)=-0.40$, $p=.092$; DDL: $r_\tau(15)=-0.59$, $p=.006$) in HL and DDL condition. Furthermore, a higher normalized power was associated with a higher IS in both conditions (HL: $r_\tau(15)=0.42$, $p=.074$; DDL: $r_\tau(15)=0.56$, $p=.008$). Interestingly, the correlations had smaller effects and were only significant by trend in the HL condition, which points towards an entraining effect of the DDL stimulation (cf. *Figure S5*).

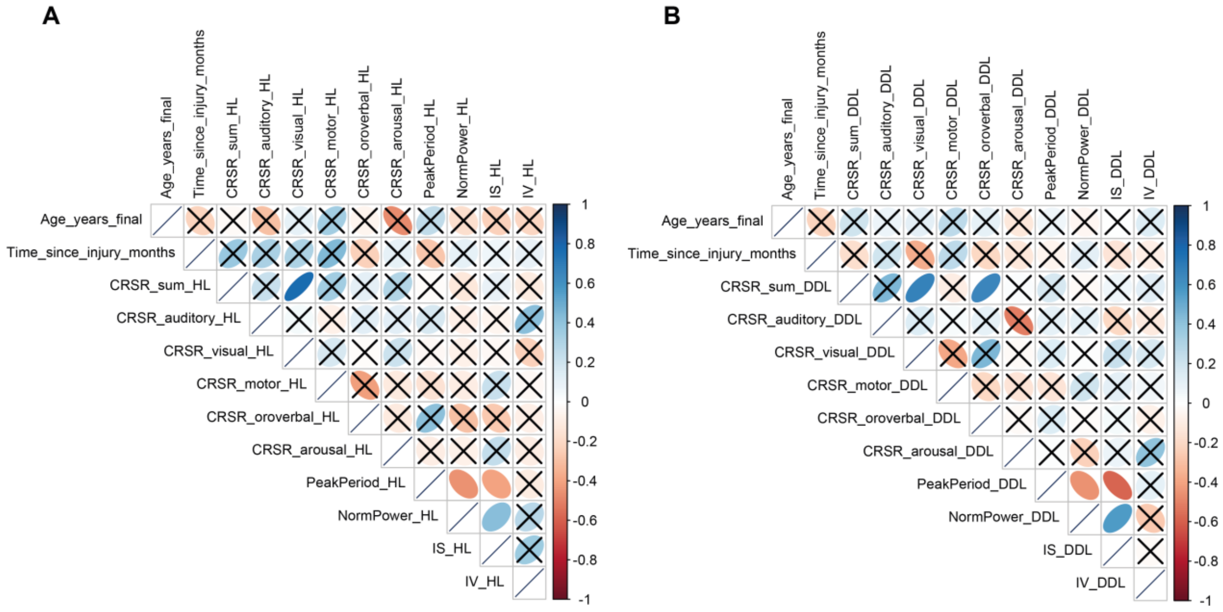

Figure S5. **Correlation matrix separately for HL (A) and DDL (B) condition.** Positive correlations are displayed in blue and negative correlations in red. Color intensity and the size of the circle are proportional to the correlation coefficients (Kendall's Tau). Crosses indicate correlations with p > 0.1. Statistics for significant correlations are mentioned in the text above. Abbreviations: *HL* habitual light, *DDL* dynamic daylight, *CRSR* Coma Recovery Scale Revised, *IS* interdaily stability, *IV* intradaily variability.

First vs. Second Recording Week

For the following analyses, we compared temperature parameters between first vs. second recording week without taking condition (HL, DDL) into account. Comparisons between time points revealed no significant difference between first and second recording week when looking at period length ($F_{ATS}(1)=0.47$, $p=.247$, $RTE_{first}=.47$, $RTE_{second}=.53$), power ($F_{ATS}(1)=0.14$, $p=.355$, $RTE_{first}=.49$, $RTE_{second}=.51$), interdaily stability ($F_{ATS}(1)=0.17$, $p=.339$, $RTE_{first}=.48$, $RTE_{second}=.52$) and intradaily variability ($F_{ATS}(1)=2.07$, $p=.075$, $RTE_{first}=.43$, $RTE_{second}=.57$) of the patient's temperature rhythm. Likewise, the CRS-R sum score did not differ between first and second recording week ($F_{ATS}(1)=1.47$, $p=.113$, $RTE_{first}=.46$, $RTE_{second}=.54$).

Additional Analyses: ECG Data

While we recorded temperature variations for the entire duration (i.e. one week) of each condition, ECG recordings were limited to shorter recording durations (i.e. due the battery of the ambulatory ECG devices not lasting longer). More specifically, ECG data were only acquired at

the beginning of each study condition (i.e. starting at the first or second day of the study protocol with a minimal recording duration of 1.5 days). Thus, we (i) only measured the immediate effect of light stimulation on the patients' cardiac activity, and (ii) were not able to investigate circadian variations, because this requires data acquired across multiple days. Thus, we analysed diurnal variations rather than circadian patterns of the patients' cardiac activity.

ECG Analyses

To explore diurnal variations in patients' heart rate (HR) and heart rate variability (HRV) in the different lighting conditions, we used continuous 24-h ECG data from each patient, and divided the ECG recording into periods of clear daytime (i.e. forenoon: 8am-2pm, afternoon: 2pm-8pm) and nighttime (i.e. 11pm-5am). Periods with conditions of twilight (i.e. dawn: 5-8am, dusk: 8-11pm) were excluded from the analyses. While HR indicates the average time interval between adjacent heartbeats (i.e. interbeat interval [IBI]), HRV quantifies the variability in these time intervals.

HRV analyses were conducted in ANSLAB 2.6 [2]. From a total of 18 patients with DOC, six patients had to be excluded from the analyses due to severe cardiac arrhythmias. Thus, we included data of twelve patients in the following analyses.

We analysed patients' IBI and patients' HRV in the time (i.e. root mean square of successive differences between adjacent heartbeats [RMSSD]) and frequency domain (i.e. very low [VLF; 0.003-0.04 Hz], low [LF; 0.04-0.15 Hz] and high frequency band [HF; 0.15-0.4 Hz]). For details on the parameters and preprocessing of ECG data please refer to Angerer et al.[3] where we examined diurnal variations of HR and HRV irrespective of light conditions in a larger sample of patients with DOC).

Statistical Analyses

For the analyses of differences in ECG parameters (i.e. IBI, RMSSD, VLF, LF, HF) between lighting conditions (i.e. within-subjects factor; HL and DDL) and different times of day (i.e. within-subjects factor; forenoon, afternoon, night) we used the "nparLD" package available for R [4]. As the analyses were exploratory, the significance level was set to $\alpha = .05$ (two-sided). For details on the package and statistics, please refer to the main manuscript.

Results

Analyses did not reveal a significant effect for *lighting condition* and the *lighting condition* × *time* interaction in any of the HRV variables (cf. *Table S4*). The main effect for *time* was significant for the VLF ($F_{ATS}(2)=4.32$, $p=.032$) and LF ($F_{ATS}(2)=5.01$, $p=.017$) band. However, post-hoc comparisons of VLF between times of day did not yield significance anymore after correcting for multiple comparisons (forenoon vs. afternoon: $F_{ATS}(1)=0.33$, $p=.567$, forenoon vs. night: $F_{ATS}(1)=2.39$, $p=.183$, afternoon vs. night: $F_{ATS}(1)=4.02$, $p=.135$). When looking at the LF band, patients showed a significantly higher LF during the night as compared to fore- ($F_{ATS}(1)=6.31$, $p=.018$) and afternoon ($F_{ATS}(1)=10.91$, $p=.003$). No significant differences were found in LF during forenoon as compared to afternoon ($F_{ATS}(1)=1.1$, $p=.295$). Thus, our results show that DDL stimulation did not have an immediate effect on patients' cardiac activity and its' diurnal pattern. Future studies should consider looking at ECG data at the end (not at the beginning) of the light exposure as a longer-lasting DDL exposure, especially in such chronic conditions, might be necessary for such an effect to be observed.

Table S4.
Anova-type statistic (ATS) of the different HRV parameters separately for the main effects "lighting condition" (i.e. habitual light, dynamic daylight) and "time" (i.e. forenoon [8am-2pm], afternoon [2pm-8pm], night [11pm-5am]), and the "lighting condition" × "time" interaction (N=12).

Variable	Lighting Condition	Time	Lighting Condition × Time
Interbeat interval	$F_{ATS}(1)=0.22$, $p=.642$<br>$RTE_{HL}=.49$<br>$RTE_{DDL}=.51$	$F_{ATS}(1.51)=0.80$, $p=.417$<br>$RTE_{Forenoon}=.47$<br>$RTE_{Afternoon}=.51$<br>$RTE_{Night}=.52$	$F_{ATS}(1.66)=1.27$, $p=.277$<br>$RTE_{HL:Forenoon}=.44$<br>$RTE_{HL:Afternoon}=.53$<br>$RTE_{HL:Night}=.50$<br>$RTE_{DDL:Forenoon}=.50$<br>$RTE_{DDL:Afternoon}=.50$<br>$RTE_{DDL:Night}=.53$
RMSSD	$F_{ATS}(1)=0.45$, $p=.503$<br>$RTE_{HL}=.48$<br>$RTE_{DDL}=.52$	$F_{ATS}(1.62)=0.25$, $p=.733$<br>$RTE_{Forenoon}=.49$<br>$RTE_{Afternoon}=.50$<br>$RTE_{Night}=.51$	$F_{ATS}(1.75)=1.79$, $p=.172$<br>$RTE_{HL:Forenoon}=.49$<br>$RTE_{HL:Afternoon}=.49$<br>$RTE_{HL:Night}=.46$<br>$RTE_{DDL:Forenoon}=.49$<br>$RTE_{DDL:Afternoon}=.50$<br>$RTE_{DDL:Night}=.57$
Very low frequency	$F_{ATS}(1)=0.76$, $p=.384$<br>$RTE_{HL}=.48$<br>$RTE_{DDL}=.52$	$F_{ATS}(1.16)=4.32$, $p=.032$<br>$RTE_{Forenoon}=.46$<br>$RTE_{Afternoon}=.47$<br>$RTE_{Night}=.57$	$F_{ATS}(1.89)=0.09$, $p=.899$<br>$RTE_{HL:Forenoon}=.44$<br>$RTE_{HL:Afternoon}=.45$<br>$RTE_{HL:Night}=.55$<br>$RTE_{DDL:Forenoon}=.48$<br>$RTE_{DDL:Afternoon}=.48$<br>$RTE_{DDL:Night}=.59$
Low frequency	$F_{ATS}(1)=0.85$, $p=.357$<br>$RTE_{HL}=.48$<br>$RTE_{DDL}=.52$	$F_{ATS}(1.28)=5.01$, $p=.017$<br>$RTE_{Forenoon}=.45$<br>$RTE_{Afternoon}=.47$<br>$RTE_{Night}=.58$	$F_{ATS}(1.96)=0.23$, $p=.787$<br>$RTE_{HL:Forenoon}=.42$<br>$RTE_{HL:Afternoon}=.45$<br>$RTE_{HL:Night}=.56$<br>$RTE_{DDL:Forenoon}=.48$<br>$RTE_{DDL:Afternoon}=.49$<br>$RTE_{DDL:Night}=.59$
High frequency	$F_{ATS}(1)=0.62$, $p=.432$<br>$RTE_{HL}=.48$<br>$RTE_{DDL}=.52$	$F_{ATS}(1.34)=0.33$, $p=.633$<br>$RTE_{Forenoon}=.48$<br>$RTE_{Afternoon}=.52$<br>$RTE_{Night}=.50$	$F_{ATS}(1.82)=1.09$, $p=.332$<br>$RTE_{HL:Forenoon}=.46$<br>$RTE_{HL:Afternoon}=.51$<br>$RTE_{HL:Night}=.46$<br>$RTE_{DDL:Forenoon}=.50$<br>$RTE_{DDL:Afternoon}=.52$<br>$RTE_{DDL:Night}=.54$

Significant *p*-values (i.e. $p < .05$) are marked in grey. Abbreviations: RMSSD = root mean square of successive differences between adjacent heartbeats.

References

1. CIES026:2018. *CIE System for Metrology of Optical Radiation for ipRGC-Influenced Responses to Light.* CIE; 2018.
2. Blechert J, Peyk P, Liedlgruber M, Wilhelm FH. ANSLAB: Integrated multichannel peripheral biosignal processing in psychophysiological science. Behav Res Methods. 2016; 48 (4): 1528-1545.
3. Angerer M, Wilhelm FH, Liedlgruber M, et al. Does the Heart Fall Asleep?-Diurnal Variations in Heart Rate Variability in Patients with Disorders of Consciousness. Brain Sci. 2022; 12 (3): 375.
4. Noguchi K, Gel YR, Brunner E, Konietschke F. nparLD: An R Software Package for the Nonparametric Analysis of Longitudinal Data in Factorial Experiments. J Stat Softw. 2012; 50 (12): 1-23.
